# A new species of Elachista Treitschke, 1833 (Lepidoptera, Elachistidae, Elachistinae) from China, with identification keys to the Asian species of the *Elachistasaccharella* species group

**DOI:** 10.3897/zookeys.1068.70807

**Published:** 2021-11-03

**Authors:** Virginijus Sruoga

**Affiliations:** 1 Life Sciences Centre of Vilnius University, Saulėtekio str. 7, LT-10257 Vilnius, Lithuania Life Sciences Centre of Vilnius University Vilnius Lithuania

**Keywords:** Asia, Henan, microlepidoptera, mining moths, morphology, taxonomy

## Abstract

A new species, *Elachistaolekarsholti***sp. nov.**, is described from Henan, China. The habitus and male genitalia are diagnosed and illustrated in detail. This is the first record of the *Elachistasaccharella* species group in China. Identification keys to the Asian species of *Elachistasaccharella* species group, based on male and female genitalia, are provided.

## Introduction

Genus *Elachista* was established by Treitschke (1833) for the type species *E.bifasciella* Treitschke, 1833. It is the most species-rich genus within the grass miner moth subfamily Elachistinae Bruand, 1850 (family Elachistidae Bruand, 1850) and currently includes about 710 described species distributed worldwide (Kaila 2019). The current concept of *Elachista* is based on the phylogenetic studies by Kaila (1999a) and Kaila and Sugisima (2011). The genus is now considered to comprise four subgenera: *Dibrachia* Sinev & Sruoga, 1992; *Hemiprosopa* Braun, 1948; *Aphelosetia* Stephens, 1834 and *Elachista* Treitschke, 1833 (Kaila 1999a). A comprehensive illustrated account of the morphology of *Elachista* is presented by Kaila (1999a) and Kaila and Sugisima (2011).

China is one of the mega-diversity countries of the world (Brooks et al. 2006). However, the *Elachista* species of China are almost unknown. In the recent catalogue of Elachistinae of the World (Kaila 2019), only four species from China were listed: *Elachistacinereopunctella* (Haworth, 1828); *E.gleichenella* (Fabricius, 1781); *E.tinctella* Sinev & Sruoga, 1995 and *E.utonella* Frey, 1856.

In the present paper, a new species of the *Elachista* is described from Henan, China. The new species is very close to species of the *Elachistaalbrechti-heteroplaca* species group (cf. Kaila 1998). This group is defined by uncus lobes being twisted basally and round apically, the basal arms of gnathos being strongly melanised, the very large juxta lobes bearing scale-like setae and valva being distally dilated and bilobed (Sugisima and Kaila 2005). Recently, in his World Catalogue of Elachistinae, Kaila (2019) merged the *Elachistaalbrechti, heteroplaca* and *solena* species groups into the *E.saccharella* species group. Synapomorphies for this group include: 1) the forewing with vein M2; 2) anterior margin of tegumen dorsomedially meeting the posterior margin (Kaila and Sugisima 2011); the latter also occurs in the *E.freyerella* species group. It also should be noted that M2 in the forewing is of quite homoplastic character, so the diagnostic value of this is very limited. *Elachistasaccharella* species group now comprise 24 described and one described, but not named species (Table 1), which are distributed in Americas, Asia, Australia and New Guinea (Kaila 2019).

**Table 1.** Species and distribution of the *Elachistasaccharella* species group.

***Elachista* species**	**Distribution**	**Notes**	**References**
*E.albrechti* Kaila, 1998	Nepal	Male only	Kaila 1998
*E.heteroplaca* Meyrick, 1934	India	Male only	Meyrick 1934; Kaila 1998
*E.lorigera* (Meyrick, 1921)	Indonesia	Female only	Meyrick 1921; Kaila 1998
*E.picroleuca* (Meyrick, 1921)	Indonesia	The holotype is without abdomen	Meyrick 1921; Kaila 2019
*E.oryx* Sruoga & Kaila, 2019	Thailand	Male only	Sruoga et al. 2019
*E.pellineni* Sruoga & Kaila, 2019	Thailand	Male and female	Sruoga et al. 2019
*E.capricornis* Sruoga & Kaila, 2019	Thailand	Male only	Sruoga et al. 2019
*E.phichaiensis* Sruoga & Kaila, 2019	Thailand	Male only	Sruoga et al. 2019
species Nr. *VS3/29.03.19*	Thailand	Described, but not named; female only	Sruoga et al. 2019
*E.olekarsholti* sp. nov.	China	Male only	Present study
*E.canis* Parenti, 1983	Japan; Russian Far East	Male and female	Parenti 1983; Sinev and Sruoga 1997; Sugisima and Kaila 2005
*E.planicara* Kaila, 1998	Japan; Russian Far East	Male and female	Kaila 1998; Sugisima and Kaila 2005
*E.sasae* Sinev & Sruoga, 1995	Japan; Russian Far East	Male and female	Sinev and Sruoga 1995; Sugisima and Kaila 2005
*E.griseola* Diakonoff, 1955	New Guinea	Male only	Diakonoff 1955; Kaila 2019
*E.ignicolor* Kaila, 2011	Australia	Male and female	Kaila 2011, 2019
*E.solena* (Bradley, 1974)	New Guinea	Male only	Bradley 1974; Kaila 1999a, 2019
*E.angularis* (Braun, 1918)	USA	Male and female	Braun 1918; Kaila 1999b
*E.brachyelytrifoliella* Clemens, 1864	USA	Male and female	Clemens 1864; Kaila 1999b
*E.dulcinella* Kaila, 1999	USA	Male and female	Kaila 1999b
*E.hedionella* Kaila, 1999	USA	Female only	Kaila 1999b
*E.helodella* Kaila, 1999	Canada; USA	Male and female	Kaila 1999b
*E.saccharella* (Busck, 1934)	Cuba; Ecuador; Peru; USA	Male and female	Busck 1934; Kaila 1999b; White et al. 2007; Sruoga 2010; Kaila 2019
*E.suavella* Kaila, 1999	USA	Male and female	Kaila 1999b
*E.uniolae* Kaila, 1999	USA	Female only	Kaila 1999b
*E.phiala* Sruoga, 2010	Ecuador	Female only	Sruoga 2010

Asian species of the group are still poorly known, but recent discoveries of four new species from Thailand (Sruoga et al. 2019) suggest that real diversity is likely much higher. For the taxonomic keys, all known Asian species of the *Elachistasaccharella* species group are included.

## Materials and methods

Adult specimens were examined externally using MBS-10 and Euromex Stereo Blue stereomicroscopes. The forewing length was measured along the costa from wing base to the apex of the terminal fringe scales. For a wingspan, the forewing length was doubled and thorax width added. The width of the head was measured between the inner edges of the antennal bases. Genitalia were prepared following the standard method described by Robinson (1976) and Traugott-Olsen and Nielsen (1977). The genitalia were studied and some morphological structures were photographed in glycerol before permanent slide-mounting in Euparal. The male genital capsule was stained with fuchsin and the abdominal pelt with chlorazol black (Direct Black 38/Azo Black). The genital morphology was examined using a Novex B microscope. The photographs of adults were taken using a Leica S6D stereomicroscope and Leica DFC290 digital camera. The photographs of genitalia were made using a Leica DM2500 microscope and a Leica DFC420 digital camera. The descriptive terminology of morphological structures follows Traugott-Olsen and Nielsen (1977); Kaila (1999a, 2011) and Kristensen (2003).

### Abbreviations for repositories

**NKU** Insect Collection of Nankai University, Tianjin, China

**ZMUC** Zoological Museum, University of Copenhagen, Denmark

## Taxonomy

### Key to the Asian species of *Elachistasaccharella* species group based on male genitalia

[males of *E.lorigera, E.picroleuca* and *E.* species Nr. *VS3/29.03.19* are unknown]

**Table:** 

1	Valva distally bilobed (Kaila 1998, figs. 2 and 14; Sruoga et al. 2019, figs. 16, 26, 42 and 53; this paper, Fig. 4)	**2**
–	Valva distally not bilobed (Sugisima and Kaila 2005, figs. 2, 6, 11 and 19)	**8**
2	Valva distally with long, strongly sclerotised spine (Kaila 1998, fig. 2; Sruoga et al. 2019, figs. 16, 26 and 42)	**3**
–	Valva distally without long, strongly sclerotised spine (Kaila 1998, fig. 14; Sruoga et al. 2019, fig. 53; this paper, Fig. 4)	**6**
3	Spine of valva strongly curved, S-shaped (Sruoga et al. 2019, fig. 42)	***E.capricornis***
–	Spine of valva straight (Kaila 1998, fig. 2; Sruoga et al. 2019, figs. 16 and 26)	**4**
4	Digitate process short, as long as wide at base, triangular, devoid of setae (Sruoga et al. 2019, fig. 16)	***E.oryx***
–	Digitate process long, more than 6 times longer than wide, apically with few setae (Kaila 1998, figs. 2 and 4; Sruoga et al. 2019, fig. 26)	**5**
5	Digitate process about twice shorter than spine of valva (Kaila 1998, figs. 2 and 4)	***E.heteroplaca***
–	Digitate process as long as spine of valva (Sruoga et al. 2019, fig. 26)	***E.pellineni***
6	Digitate process strongly dilated apically; spinose knob of gnathos indentated (Kaila 1998, figs. 14 and 16)	***E.albrechti***
–	Digitate process not dilated apically; spinose knob of gnathos not indentated (Sruoga et al. 2019, fig. 53; this paper, Figs 4 and 12)	**7**
7	Digitate process more than ten times as long as wide (Sruoga et al. 2019, fig. 53)	***E.phichaiensis***
–	Digitate process about three times as long as wide (this paper, Fig. 4)	***E.olekarsholti* sp. nov.**
8	Digitate process absent (Sugisima and Kaila 2005, fig. 19)	***E.planicara***
–	Digitate process present (Sugisima and Kaila 2005, figs. 2, 6 and 11)	**9**
9	Costa of valva with hump at 1/3 from the base; phallus as long as valva (Sugisima and Kaila 2005, figs. 2 and 6)	***E.canis***
–	Costa of valva with hump at 2/3 from the base; phallus 4/5 length of valva (Sugisima and Kaila 2005, fig. 11)	***E.sasae***

### Key to the Asian species of *Elachistasaccharella* species group based on female genitalia

[females of *E.albrechti, E.heteroplaca*, *E.picroleuca, E.oryx, E.capricornis, E.phichaiensis* and *E.olekarsholti* sp. nov. are unknown]

**Table:** 

1	Corpus bursae without signum (Kaila 1998, fig. 11)	***E.planicara***
–	Corpus bursae with signum (Kaila 1998, fig. 7; Sugisima and Kaila 2005, figs. 7 and 12; Sruoga et al. 2019, figs. 37 and 62)	**2**
2	Signum boomerang-shaped, forming an angle of about 120 degrees (Sruoga et al. 2019, fig. 37)	***E.pellineni***
–	Signum not boomerang-shaped, straight or weakly curved, forming an angle less than 40 degrees (Kaila 1998, fig. 7; Sugisima and Kaila 2005, figs. 7 and 12; Sruoga et al. 2019, fig. 62)	**3**
3	Antrum cup-shaped, anteriorly abruptly narrowing into ductus bursae (Kaila 1998, fig. 7; Sugisima and Kaila 2005, fig. 7)	**4**
–	Antrum gradually narrowing towards ductus bursae (Sugisima and Kaila 2005, fig. 12; Sruoga et al. 2019, fig. 61)	**5**
4	Apophysis anterioris 3/5–2/3 as long as apophysis posterioris; antrum with dense internal spines (Sugisima and Kaila 2005, fig. 7)	***E.canis***
–	Apophysis anterioris twice shorter than apophysis posterioris; antrum without internal spines (Kaila 1998, fig. 7)	***E.lorigera***
5	Colliculum width about 0.2 times width of ostium bursae; corpus bursae with internal spines (Sugisima and Kaila 2005, fig. 12)	***E.sasae***
–	Colliculum width about 0.8 times width of ostium bursae; corpus bursae without internal spines (Sruoga et al. 2019, figs. 61 and 62)	***E.* species Nr. *VS3/29.03.19***

#### Elachista olekarsholti sp. nov.

Taxon classificationAnimaliaLepidopteraElachistidae

87DC08D7-BC43-5354-A24E-3AB6E1772C85

http://zoobank.org/DD59D982-4E63-4797-B541-F9736A0D47AE

Figures 1–3 , 4–8 , 9–12

##### Material examined.

***Holotype.*** CHINA • ♂; Henan Prov.[ince], Tongbai; 300 m alt.; 11–13 Sep. 2000; O. Karsholt leg.; NKU VS501. ***Paratype.*** CHINA • 1 ♂; same label as holotype; ZMUC VS502.

##### Diagnosis.

*Elachistaolekarsholti* belongs to the *E.saccharella* species group. It is a small, dark-coloured species with indistinct wing markings and a dorsoventrally flattened head. In wing pattern and male genitalia, the new species is most similar to *E.albrechti* Kaila, 1998, known from Nepal. The main differences between *E.albrechti* (cf. Kaila 1998) and *E.olekarsholti* are: (1) spinose knob of gnathos very long and narrow in *E.olekarsholti*, in *E.albrechti*, it is club-shaped, with large distal dilation; (2) digitate process in *E.olekarsholti* is short and narrow, in *E.albrechti*, it is strongly dilated; (3) saccus in *E.olekarsholti* very short, whereas it is three times longer than wide in *E.albrechti*; (4) phallus in *E.olekarsholti* strongly curved beyond the middle, with cornutus, in *E.albrechti*, it is strongly curved before the middle, without cornutus.

##### Male

**Figures 1–3.**
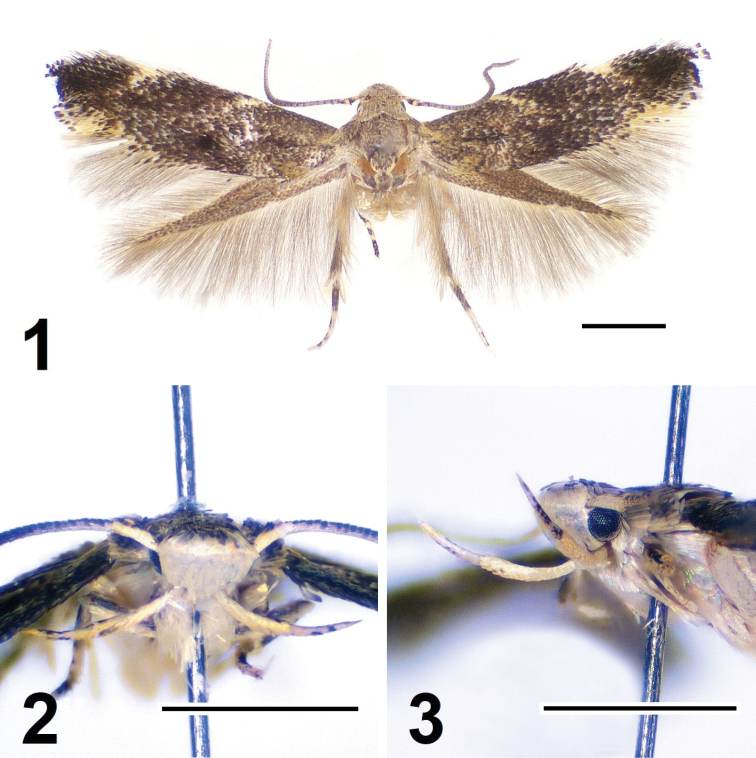
*Elachistaolekarsholti* sp. nov. **1** male adult, holotype **2** same, head, frontal view **3** head, paratype, lateral view. Scale bars: 1 mm.

(Figs 1–3). Forewing length 3.5–3.6 mm; wingspan 7.7–7.9 mm (n = 2). **Head**: frons shiny, creamy white; vertex whitish-brown; neck tuft greyish-brown; labial palpus upwards curved, diverging, about 1.7 times as long as width of head, whitish-creamy, distal part of second and third segment with few dark brown scales; scape creamy white below, with few dark brown scales above, without pecten; flagellum blackish-brown above, weakly annulated with paler rings, basal part creamy white below. Thorax and tegula greyish-brown, mottled with dark brown tipped scales. **Forewing**: ground colour blackish-brown, basal part slightly paler, intermixed with few rusty scales; indistinctly delimited oblique whitish-creamy streak from 1/3 of costa to fold where there is a small group of raised black scales; indistinct whitish creamy spot at 2/3 length of costa and similar one on dorsum just before it; fringe scales brownish-grey, fringe line brownish-black. Hind-wing brownish-grey, with fringe concolorous.

**Female.** Unknown.

**Figures 4–8.**
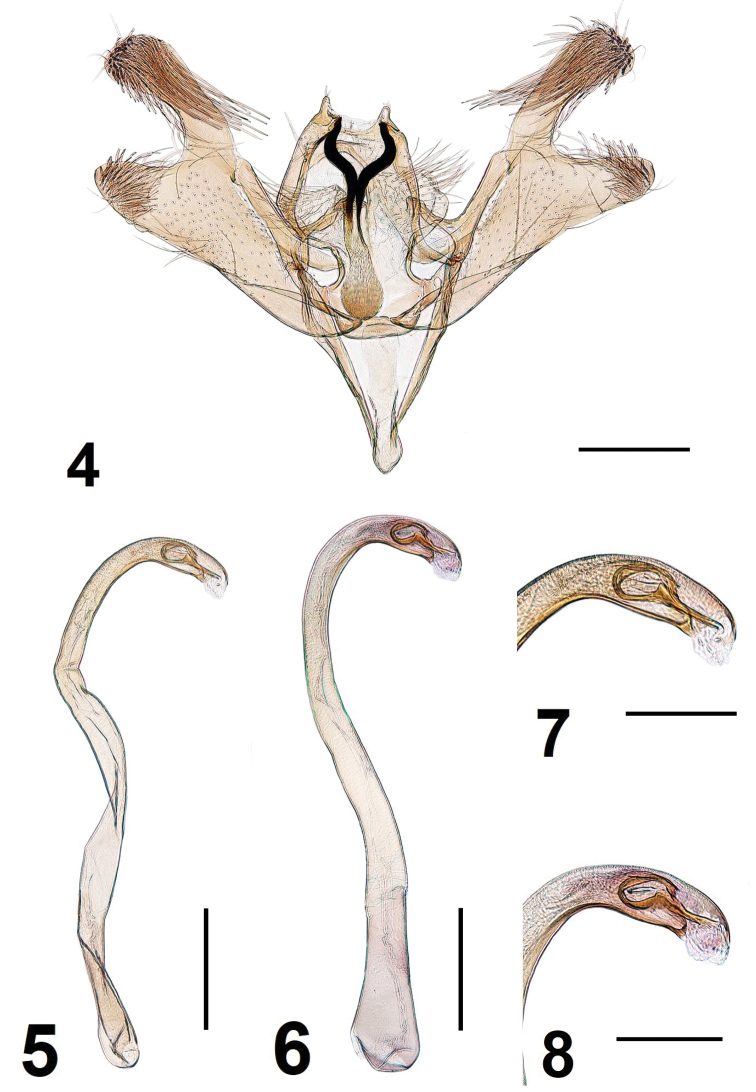
*Elachistaolekarsholti* sp. nov. **4** general view of male genitalia (phallus removed), holotype **5** phallus, holotype **6** phallus, paratype **7** apical part of phallus, holotype **8** apical part of phallus, paratype. Scale bars: 0.1 mm.

**Figures 9–12.**
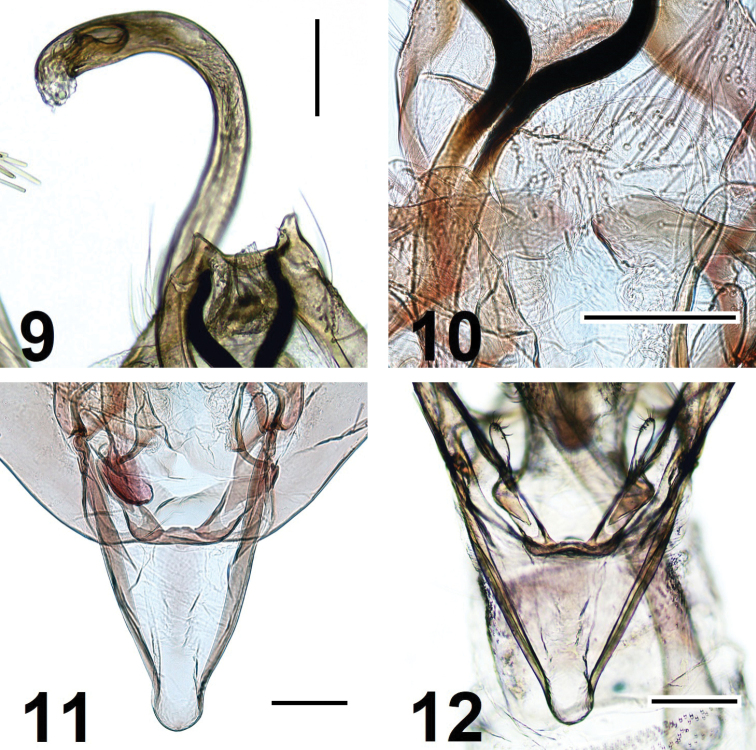
*Elachistaolekarsholti* sp. nov. **9** uncus lobes and distal part of phallus, paratype, in glycerol before permanent mounting in Euparal **10** apical part of juxta lobe, paratype **11** vinculum and saccus, paratype **12** same, in glycerol before permanent mounting in Euparal. Scale bars: 0.1 mm.

**Male genitalia** (Figs 4–12). Uncus lobes very small, triangular-shaped, apex with few tiny setae. Basal arms of gnathos very long, heavily melanised and strongly bent towards posterior direction, apically fused; spinose knob about two times as long as wide, apically widened. Costa of valva almost straight; basal fold of costa meets distal fold at 1/3 from base. Cucullus medially deeply incised, thus divided into two lobes: triangular lobe where sacculus meets cucullus and another longer distal lobe. Digitate process short and slender, three times as long as its width, distally with few short setae. Juxta lobes large, about 1/4 length of valva, mesially somewhat produced, medial incision between juxta lobes very short (Figs 4 and 10), distal margin medially slightly concave, ventral surface with short setae medially and long setae laterally. Vinculum with broad median ridge, tapered to short and broad saccus. Phallus about 1.6 length of valva, twisted and strongly curved at basal 1/2 and 4/5; vesica with group of minute spines and long folded cornutus.

##### Biology.

Unknown.

##### Flight period.

Based on the specimens available, adults fly in September.

##### Distribution.

So far, this species is known only from east-central China.

##### Etymology.

The new species is named in honour of Ole Karsholt (Copenhagen, Denmark) who collected the type specimens.

##### Remarks.

The phallus of the holotype is slightly distorted during slide mounting and, therefore, looks somewhat skewed in Fig. 5.

## Supplementary Material

XML Treatment for Elachista olekarsholti
